# Impact of a Transformational Leadership Program for Nurse Managers on Nurses' Job Satisfaction and Work Environment in Saudi Arabian Primary Healthcare Centers: A Quasi-Experimental Study  

**DOI:** 10.12688/f1000research.188950.1

**Published:** 2026-08-27

**Authors:** Alrashidi Nasser Ayidh M, Tukimin bin Sansuwito

**Affiliations:** 1 Faculty of Nursing, Lincoln University College, Petaling Jaya, Selangor, Malaysia

**Keywords:** Transformational leadership, job satisfaction, work environment, primary healthcare, nurses, quasi-experimental study, Saudi Arabia, leadership development.

## Abstract

**Background:**

Transformational leadership has been associated with improved organizational performance and employee well-being. However, evidence on structured transformational leadership program in primary healthcare settings remains limited, particularly in Saudi Arabia. This study evaluated the effectiveness of a transformational leadership program in improving nurses’ job satisfaction and perceptions of the work environment in primary healthcare centers in Medina, Saudi Arabia.

**Methods:**

A quasi-experimental pre-test and post-test study with a control group was conducted among 104 nurses working in Ministry of Health primary healthcare centers in Medina, Saudi Arabia. Participants were assigned to an intervention group (n = 52) or a control group (n = 52). The intervention comprised a structured transformational leadership program delivered to nurse managers over two weeks. Job satisfaction was assessed using the Minnesota Satisfaction Questionnaire (MSQ), while the work environment was assessed using the Areas of Worklife Survey (AWS). Within-group changes were examined using paired-samples t-tests, and analysis of covariance (ANCOVA) was used to compare post-intervention outcomes between groups after adjustment for baseline scores.

**Results:**

Significant improvements were observed in the intervention group compared with the control group across all study outcomes. After adjustment for baseline scores, ANCOVA demonstrated significant intervention effects on overall job satisfaction (partial η ^2^ = 0.883), intrinsic job satisfaction (partial η ^2^ = 0.910), extrinsic job satisfaction (partial η ^2^ = 0.689), and work environment (partial η ^2^ = 0.747) (all p < 0.001).

**Conclusions:**

A structured transformational leadership program delivered to nurse managers over two weeks was associated with significant improvements in nurses’ job satisfaction and perceptions of the work environment in primary healthcare centers. Leadership development may provide a practical organizational strategy for strengthening nurses’ workplace well-being and improving work environments in primary healthcare settings.

## Introduction

Primary healthcare (PHC) is a cornerstone of healthcare systems, providing accessible, comprehensive, and community-oriented care across the continuum of health services. Nurses constitute a substantial proportion of the healthcare workforce and play central roles in direct patient care, health promotion, disease prevention, patient education, care coordination, and quality improvement. Maintaining a motivated and satisfied nursing workforce and a supportive work environment is therefore important for sustaining healthcare quality and organizational performance. ^1^ ^,^ ^2^ In Saudi Arabia, the work environment of nurses in PHC centers is particularly relevant, as a national study involving 519 nurses identified variability in perceptions of staffing and resources and other dimensions of the nursing practice environment. ^3^

Job satisfaction is an important indicator of nurses’ workplace well-being and organizational functioning. Higher job satisfaction has been associated with greater organizational commitment and intention to remain in the workforce, whereas lower satisfaction has been linked to workforce instability and turnover intention. ^2^ ^,^ ^4^ Nurses’ job satisfaction is influenced by multiple factors, including leadership, workload, staffing, organizational support, professional development opportunities, workplace relationships, and working conditions. ^1^ ^,^ ^2^ Evidence from Saudi Arabian PHC centers also indicates that supervision, communication, and other organizational factors are relevant to healthcare providers’ job satisfaction and intention to leave. ^9^

Leadership is a potentially modifiable organizational factor that may influence nurses’ job satisfaction and work environment. Transformational leadership emphasizes idealised influence, inspirational motivation, intellectual stimulation, and individualized consideration and seeks to motivate staff, encourage professional development, facilitate collaboration, and support organizational change. ^5^ Previous research has associated transformational leadership with nurses’ job satisfaction and positive workplace outcomes. For example, Boamah et al. ^6^ found that transformational leadership by nurse managers was positively related to workplace empowerment, which in turn was associated with higher nurses’ job satisfaction and more favourable patient safety outcomes. Systematic review evidence has likewise indicated that transformational leadership can positively influence nurses’ work environments, with job satisfaction, organizational commitment, and empowerment acting as important pathways. ^7^

The relationship between nurse managers’ leadership and staff nurses’ outcomes is particularly relevant because nurse managers directly influence nurses’ daily working conditions, communication, support, and opportunities for participation. Uslu Sahan and Terzioglu, ^8^ for example, found that transformational leadership practices of nurse managers were associated with staff nurses’ job satisfaction and organizational commitment in a study involving 17 nurse managers and 136 staff nurses. However, much of the available evidence remains observational or correlational, limiting conclusions regarding the effects of structured leadership development interventions. Recent intervention research has demonstrated that leadership development programs can improve the leadership knowledge and practices of nurse leaders. Emam et al., ^10^ for example, reported significant improvements in leadership knowledge and practices following a six-week leadership development program for head nurses. Nevertheless, evidence remains limited regarding whether a structured transformational leadership program delivered specifically to nurse managers can produce measurable improvements in staff nurses’ job satisfaction and perceptions of their work environment.

This gap is particularly relevant in Saudi Arabia, where ongoing healthcare reforms under Saudi Vision 2030 emphasize healthcare quality, patient-centered care, workforce development, and organizational performance. PHC centers represent an important setting for these reforms, and evidence from Eastern Saudi Arabia has highlighted the importance of organizational factors, including supervision and communication, for healthcare providers’ satisfaction. ^9^ Developing nurse managers’ leadership capabilities may therefore represent a potentially useful organizational strategy for strengthening nurses’ workplace experiences. Although transformational leadership has been examined among Saudi nursing leaders and supervisors, intervention-based evidence from Saudi Arabian PHC settings remains limited. ^4^ ^,^ ^10^

Accordingly, this study aimed to determine whether a structured transformational leadership program delivered to nurse managers was associated with improvements in staff nurses’ job satisfaction and perceptions of the work environment in primary healthcare centers in Medina, Saudi Arabia, compared with usual leadership practice. The study specifically assessed changes in overall, intrinsic, and extrinsic job satisfaction and perceptions of the work environment following the intervention.

## Methods

### Study design and setting

This quasi-experimental study employed a pre-test and post-test control group design to evaluate the effectiveness of a structured transformational leadership program delivered to nurse managers on staff nurses’ job satisfaction and perceptions of the work environment. The study was conducted in seven government-operated Ministry of Health primary healthcare centers (PHCs) in Medina, Saudi Arabia. The participating PHCs were Al-Hijrah Health Center, Wa’irah Health Center, Al-Safiyyah Health Center, the Health Surveillance Center at Prince Mohammed bin Abdulaziz International Airport, Al-Daithah Health Center, Bab Jibreel Health Center, and Al-Salsalah Health Center. Four PHCs comprised the intervention group and three comprised the control group. The intervention was delivered to nurse managers in the intervention PHCs, while outcomes were assessed among staff nurses working under their supervision.

### Participants and sample size

The target population comprised registered nurses employed at the seven participating PHCs, with a total population of 142 nurses. The required sample size was calculated using a 95% confidence level and a 5% margin of error for the finite study population, resulting in a required sample of 104 nurses. Convenience non-probability sampling was used to recruit eligible participants until the required sample size was achieved. A total of 104 nurses were included in the study, with 52 nurses in the intervention group and 52 in the control group. All participants completed both the pre-intervention and post-intervention assessments and were included in the final analysis. Eligible participants were registered nurses employed at one of the participating PHCs who provided written informed consent. Non-nursing staff and nurses who declined to provide informed consent were excluded.

### Intervention and control conditions

The intervention group comprised four PHCs: Al-Hijrah Health Center, Wa’irah Health Center, Al-Safiyyah Health Center, and the Health Surveillance Center at Prince Mohammed bin Abdulaziz International Airport. The control group comprised three PHCs: Al-Daithah Health Center, Bab Jibreel Health Center, and Al-Salsalah Health Center. Nurse managers in the intervention PHCs received the transformational leadership program, whereas nurse managers in the control PHCs continued their routine leadership practices during the study period.

### Transformational leadership program

The transformational leadership program was developed to strengthen nurse managers’ leadership competencies and their ability to foster supportive workplace relationships, effective communication, team motivation, and a positive organizational climate. The program was based on the four core dimensions of transformational leadership: idealised influence, inspirational motivation, intellectual stimulation, and individualized consideration.

The program was delivered over two weeks and comprised five training days, with two training days during the first week and three during the second week. Each training session lasted approximately three hours. The program combined interactive lectures, facilitated group discussions, case-based learning, role-playing exercises, leadership simulations, and practical problem-solving activities. The training covered transformational leadership principles, effective communication, team motivation, conflict management, ethical leadership, decision-making, and strategies for improving workplace collaboration. Practical activities enabled nurse managers to apply transformational leadership behaviours to realistic situations encountered in primary healthcare practice.

Program evaluation included a pre-training assessment before the intervention and a post-training assessment at program completion. Participants also completed a program evaluation form and received certificates of completion. Structured observational checklists were used during practical leadership exercises to assess leadership behaviours. Following completion of the program, weekly unannounced observational visits were conducted for six months to monitor the application of transformational leadership behaviours in routine managerial practice.

### Outcome measures

The primary study outcomes were nurses’ job satisfaction and perceptions of the work environment. Outcomes were assessed using standardised self-administered questionnaires administered before the intervention and after completion of the two-week program.

### Job satisfaction

Job satisfaction was assessed using the 20-item short-form Minnesota Satisfaction Questionnaire (MSQ). The MSQ measures intrinsic, extrinsic, and overall job satisfaction using a five-point Likert scale ranging from 1 (very dissatisfied) to 5 (very satisfied). The intrinsic satisfaction scale comprises 12 items, the extrinsic satisfaction scale comprises six items, and two items assess general satisfaction. Total scores range from 20 to 100, with higher scores indicating greater job satisfaction. Previous evidence reported Cronbach’s alpha coefficients of 0.79 for intrinsic satisfaction and 0.82 for extrinsic satisfaction.

### Work environment

Perceptions of the work environment were assessed using the Areas of Worklife Survey (AWS). The AWS assesses six dimensions of the work environment: workload, control, reward, community, fairness, and values. Higher scores indicate more favourable perceptions of the work environment.

### Questionnaire pre-testing, validity and reliability

The questionnaire was pilot-tested among 25 nurses at Al-Hijrah PHC before the main study to assess the clarity and comprehensibility of the items. Content validity was assessed before the main data collection, and the proposal reported that the content validity indices exceeded the specified acceptable threshold. Internal consistency was assessed using Cronbach’s alpha, with a coefficient greater than 0.70 considered acceptable. The MSQ was selected because of its established validity and reliability and its previous use in healthcare settings, including Saudi Arabia.

### Data collection procedures

Before data collection, eligible nurses were informed about the study objectives, procedures, voluntary nature of participation, and confidentiality of the information collected. The study procedures and questionnaire administration were conducted online. Eligible nurses received the study information and an electronic informed consent form before completing the online questionnaire. Only nurses who provided informed consent were enrolled in the study **.** Baseline questionnaires were completed online by nurses in both intervention and control PHCs before nurse managers in the intervention group participated in the transformational leadership program.

Following completion of the two-week intervention, the same outcome measures were administered online to assess post-intervention changes in nurses’ job satisfaction and perceptions of the work environment. The same standardized online questionnaire administration procedures were used for participants from all participating PHCs.

Weekly unannounced observational visits were subsequently conducted for six months to monitor the implementation of transformational leadership behaviours among nurse managers in the intervention group. These observations were used to monitor intervention implementation and were not the primary assessment of nurses’ job satisfaction or work environment.

### Strategies to minimise bias

Several procedures were used to promote consistency in intervention implementation and outcome assessment. The same outcome measures were administered before and after the intervention, and standardized questionnaire administration procedures were applied across participating PHCs. Participation was voluntary, written informed consent was obtained, and confidentiality of participant responses was maintained. The control group continued routine leadership practices during the study period, providing a comparison condition for assessing changes over time. Weekly unannounced observational visits were used to monitor the implementation of the intervention following training.

### Statistical analysis

Data were analyzed using IBM SPSS Statistics version 20.0. Descriptive statistics were used to summarise participant characteristics and study variables. Continuous variables were presented as means and standard deviations, while categorical variables were presented as frequencies and percentages.

Independent-samples *t*-tests were used to compare continuous study outcomes between the intervention and control groups at baseline. Paired-samples *t*-tests were used to assess changes in overall job satisfaction, intrinsic job satisfaction, extrinsic job satisfaction, and work environment from pre-intervention to post-intervention within each group. Cohen’s *d* was calculated to quantify the magnitude of within-group changes.

Because significant baseline differences were observed between the intervention and control groups for overall, intrinsic, and extrinsic job satisfaction, as well as work environment, ANCOVA was used as the primary comparative analysis to compare post-intervention outcomes between groups while adjusting for the corresponding baseline scores. The assumption of homogeneity of regression slopes was assessed before ANCOVA. Adjusted means, *F* statistics, *p* values, and partial eta squared (partial η ^2^) were reported as measures of intervention effect and effect size.

Pearson correlation analysis was conducted to examine the relationships among intrinsic job satisfaction, extrinsic job satisfaction, work environment, and overall job satisfaction. Associations between participant characteristics and overall job satisfaction were examined using independent-samples *t*-tests for categorical variables with two groups and one-way analysis of variance (ANOVA) for variables with more than two groups. All statistical tests were two-sided, and *p* < 0.05 was considered statistically significant.

## Results

### Participant characteristics

A total of 104 registered nurses participated in the study, comprising 52 nurses in the intervention group and 52 in the control group. All participants completed both the pre-intervention and post-intervention assessments and were included in the final analysis. Participant characteristics are presented in Table 1.

**Table 1.** Participant characteristics of the study population (n = 104).

Characteristic	n (%)
**Age (years)**	
18–25	19 (18.3)
26–35	27 (26.0)
36–45	31 (29.8)
46–55	20 (19.2)
≥56	7 (6.7)
**Gender**	
Male	47 (45.2)
Female	57 (54.8)
**Nationality**	
Saudi	96 (92.3)
Non-Saudi	8 (7.7)
**Marital status**	
Single	22 (21.2)
Married	52 (50.0)
Divorced	19 (18.3)
Separated	4 (3.8)
Widowed	7 (6.7)
**Educational level**	
Diploma	39 (37.5)
Bachelor’s degree	50 (48.1)
Master’s degree	14 (13.5)
PhD	1 (1.0)
**Professional experience**	
<1 year	10 (9.6)
1–5 years	25 (24.0)
6–10 years	22 (21.2)
11–20 years	23 (22.1)
>20 years	24 (23.1)
**Role in household**	
Head of household	31 (29.8)
Breadwinner	73 (70.2)
**Monthly income (SAR)**	
0–19,999	58 (55.8)
20,000–30,000	30 (28.8)
30,000	16 (15.4)
**PHC operating hours**	
8 am–11 pm	27 (26.0)
24 hours	77 (74.0)

The largest proportion of participants was aged 36–45 years (31/104, 29.8%), followed by those aged 26–35 years (27/104, 26.0%). More than half of the participants were female (57/104, 54.8%), Saudi nationals (96/104, 92.3%), and married (52/104, 50.0%). Regarding educational attainment, 50 participants (48.1%) held a bachelor’s degree and 39 (37.5%) held a diploma. Professional experience varied, with 24 participants (23.1%) reporting more than 20 years of experience.

### Baseline comparison between intervention and control groups

Baseline study outcomes are presented in Table 2. Significant differences were observed between the intervention and control groups for all four study outcomes. The intervention group had higher baseline overall job satisfaction than the control group (56.40 ± 13.79 vs. 49.50 ± 10.35; t = 2.887, p = 0.005). Intrinsic job satisfaction was also higher in the intervention group (32.60 ± 9.34 vs. 28.44 ± 6.67; t = 2.611, p = 0.010), as was extrinsic job satisfaction (23.81 ± 5.39 vs. 21.06 ± 4.62; t = 2.791, p = 0.006). Work environment scores were likewise higher in the intervention group than in the control group (36.48 ± 8.47 vs. 32.33 ± 9.02; t = 2.421, p = 0.017). These baseline differences were taken into account in the subsequent ANCOVA analyses.

**Table 2.** Baseline comparison of study outcomes between intervention and control groups.

Outcome	Intervention Mean ± SD	Control Mean ± SD	*t*	*p* value
Overall job satisfaction	56.40 ± 13.79	49.50 ± 10.35	2.887	0.005
Intrinsic job satisfaction	32.60 ± 9.34	28.44 ± 6.67	2.611	0.010
Extrinsic job satisfaction	23.81 ± 5.39	21.06 ± 4.62	2.791	0.006
Work environment	36.48 ± 8.47	32.33 ± 9.02	2.421	0.017

### Changes in study outcomes following the intervention

Changes in study outcomes from pre-intervention to post-intervention are presented in Table 3. In the intervention group, significant improvements were observed in all four outcomes. Intrinsic job satisfaction increased from 32.60 ± 9.34 at baseline to 57.23 ± 1.72 after the intervention (mean difference = 24.63; *t* = −19.934, *p* < 0.001; Cohen’s *d* = 2.764). Extrinsic job satisfaction increased from 23.81 ± 5.39 to 38.06 ± 1.18 (mean difference = 14.25; *t* = −18.837, *p* < 0.001; Cohen’s *d* = 2.612).

**Table 3.** Changes in study outcomes before and after the intervention.

Outcome	Group	Pre-test Mean ± SD	Post-test Mean ± SD	Mean Difference	*t*	*p* value	Cohen’s *d*
**Intrinsic job satisfaction**	Intervention	32.60 ± 9.34	57.23 ± 1.72	24.63	−19.934	<0.001	−2.764
	Control	28.44 ± 6.67	27.90 ± 6.08	0.54	0.402	0.689	0.056
**Extrinsic job satisfaction**	Intervention	23.81 ± 5.39	38.06 ± 1.18	14.25	−18.837	<0.001	−2.612
	Control	21.06 ± 4.62	21.33 ± 7.58	−0.27	−0.225	0.823	−0.031
**Work environment**	Intervention	36.48 ± 8.47	52.63 ± 1.72	16.15	−13.779	<0.001	−1.911
	Control	32.33 ± 9.02	32.50 ± 7.88	−0.17	−0.112	0.911	−0.016
**Overall job satisfaction**	Intervention	56.40 ± 13.79	95.29 ± 2.46	38.88	−20.719	<0.001	−2.873
	Control	49.50 ± 10.35	49.23 ± 11.29	0.27	0.121	0.904	0.017

Work environment scores increased from 36.48 ± 8.47 to 52.63 ± 1.72 (mean difference = 16.15; *t* = −13.779, *p* < 0.001; Cohen’s *d* = 1.911). Overall job satisfaction increased from 56.40 ± 13.79 to 95.29 ± 2.46 (mean difference = 38.88; *t* = −20.719, *p* < 0.001; Cohen’s *d* = 2.873). In the control group, no statistically significant changes were observed in intrinsic job satisfaction ( *p* = 0.689), extrinsic job satisfaction ( *p* = 0.823), work environment ( *p* = 0.911), or overall job satisfaction ( *p* = 0.904). Effect sizes were negligible across all four outcomes.

### Adjusted intervention effects

Because significant baseline differences were observed for all four study outcomes, ANCOVA was used to compare post-intervention outcomes between the intervention and control groups after adjustment for baseline scores. The results are presented in Table 4. After adjustment for baseline scores, the intervention group had significantly higher post-intervention scores than the control group for overall job satisfaction, intrinsic job satisfaction, extrinsic job satisfaction, and work environment (all *p* < 0.001).

**Table 4.** ANCOVA results comparing post-intervention outcomes after adjustment for baseline scores.

Outcome	Adjusted mean (Intervention)	Adjusted mean (Control)	*F*	*p* value	Partial η ^2^
Overall job satisfaction	95.170	48.882	759.851	<0.001	0.883
Intrinsic job satisfaction	57.231	27.904	1016.011	<0.001	0.910
Extrinsic job satisfaction	37.984	21.401	224.017	<0.001	0.689
Work environment	52.480	32.654	298.502	<0.001	0.747

The adjusted mean for overall job satisfaction was 95.170 in the intervention group compared with 48.882 in the control group ( *F* = 759.851, *p* < 0.001, partial η ^2^ = 0.883). For intrinsic job satisfaction, the adjusted means were 57.231 and 27.904, respectively ( *F* = 1016.011, *p* < 0.001, partial η ^2^ = 0.910). For extrinsic job satisfaction, the adjusted means were 37.984 and 21.401 ( *F* = 224.017, *p* < 0.001, partial η ^2^ = 0.689). For work environment, the adjusted means were 52.480 and 32.654 ( *F* = 298.502, *p* < 0.001, partial η ^2^ = 0.747).

### Correlations among study variables

Pearson correlation coefficients are presented in Table 5. Significant positive correlations were observed among all study variables (all *p* < 0.001). Overall job satisfaction demonstrated a strong positive correlation with intrinsic job satisfaction ( *r* = 0.959), extrinsic job satisfaction ( *r* = 0.891), and work environment ( *r* = 0.801). Intrinsic job satisfaction was also positively correlated with extrinsic job satisfaction ( *r* = 0.726) and work environment ( *r* = 0.750). Extrinsic job satisfaction was positively correlated with work environment ( *r* = 0.742).

**Table 5.** Pearson correlation matrix among study variables.

Variable	Intrinsic job satisfaction	Extrinsic job satisfaction	Work environment	Overall job satisfaction
Intrinsic job satisfaction	1.000	0.726	0.750	0.959
Extrinsic job satisfaction	0.726	1.000	0.742	0.891
Work environment	0.750	0.742	1.000	0.801
Overall job satisfaction	0.959	0.891	0.801	1.000

### Associations between participant characteristics and overall job satisfaction

Associations between selected participant characteristics and overall job satisfaction are presented in Table 6. Educational level was significantly associated with overall job satisfaction ( *F* = 12.39, *p* < 0.001). No statistically significant associations were observed for gender ( *t* = −1.217, *p* = 0.227), age ( *F* = 1.38, *p* = 0.246), or professional experience ( *F* = 2.04, *p* = 0.094).

**Table 6.** Association between participant characteristics and overall job satisfaction.

Participant characteristic	Statistical test	Test statistic	*p* value	Interpretation
Gender	Independent-samples *t*-test	*t* = −1.217	0.227	Not significant
Age	One-way ANOVA	*F* = 1.38	0.246	Not significant
Educational level	One-way ANOVA	*F* = 12.39	<0.001	Significant
Professional experience	One-way ANOVA	*F* = 2.04	0.094	Not significant

## Discussion

This study evaluated whether a structured transformational leadership program delivered to nurse managers was associated with improvements in staff nurses’ job satisfaction and perceptions of the work environment in primary healthcare centers in Medina, Saudi Arabia. The intervention group showed significant pre- to post-intervention improvements in overall, intrinsic, and extrinsic job satisfaction and work environment, whereas no significant changes were observed in the control group. Importantly, significant baseline differences were present between the intervention and control groups for all four outcomes. After adjustment for baseline scores using ANCOVA, the intervention group continued to demonstrate substantially higher post-intervention scores across all outcomes. These findings provide intervention-based evidence supporting the potential value of transformational leadership development for nurse managers in primary healthcare settings.

The findings are broadly consistent with previous evidence linking transformational leadership with nurses’ job satisfaction and positive workplace outcomes. Gebreheat et al., ^12^ in an integrative review, identified a consistent positive relationship between transformational leadership and nurses’ job satisfaction. Similarly, Ystaas et al. ^7^ reported that transformational leadership was associated with more favourable nursing work environments and identified job satisfaction, organizational commitment, and empowerment as important outcomes associated with transformational leadership. The present findings extend this literature by examining a structured leadership intervention rather than relying solely on nurses’ perceptions of existing leadership practices. The results are also consistent with evidence that leadership development programs can strengthen leadership practices among nurse leaders. ^12^

The improvement in overall job satisfaction observed in the intervention group may reflect several mechanisms associated with transformational leadership. Transformational leadership emphasizes inspirational motivation, individualized consideration, intellectual stimulation, and idealised influence, which may encourage supportive communication, recognition, participation, professional development, and shared problem-solving. ^5^ These leadership behaviours are particularly relevant in primary healthcare settings, where nurse managers are responsible for coordinating staff, supporting teamwork, responding to workplace challenges, and maintaining effective day-to-day communication. Previous research has shown that transformational leadership practices among nurse managers are associated with staff nurses’ job satisfaction and organizational commitment. ^8^ The present study adds to this evidence by suggesting that structured development of transformational leadership competencies among nurse managers may be accompanied by improvements in staff nurses’ workplace outcomes.

Intrinsic job satisfaction demonstrated the largest adjusted intervention effect, with a partial η ^2^ of 0.910. This finding suggests that the intervention was particularly strongly associated with aspects of satisfaction related to the intrinsic experience of work. Transformational leadership may plausibly influence these experiences by providing greater recognition, encouragement, opportunities for professional development, and support for nurses’ participation in problem-solving and decision-making. However, the present design does not allow us to determine whether improvements in intrinsic satisfaction preceded or caused changes in extrinsic satisfaction or other workplace outcomes. The large effect should therefore be interpreted as an observed adjusted group difference rather than evidence of a specific causal pathway. This interpretation is consistent with previous literature linking transformational leadership with nurses’ job satisfaction and professional development. ^11^ ^,^ ^13^

The intervention was also associated with substantial improvements in extrinsic job satisfaction and perceptions of the work environment. This finding is particularly relevant because the work environment represents an important organizational context in which nurses experience workload, control, rewards, relationships, fairness, and shared values. Evidence from Saudi Arabian primary healthcare settings has highlighted the importance of organizational factors for healthcare providers’ satisfaction, while research among Saudi nurses has also identified leadership as an important factor associated with satisfaction and intention to remain in the organisation. ^4^ ^,^ ^9^ In addition, research examining nursing practice environments in Saudi Arabian PHC centers indicates the importance of organizational conditions and adequate support for nurses’ workplace experiences. ^3^ The present findings therefore suggest that leadership development may be relevant not only to individual attitudes toward work but also to perceptions of the broader work environment.

The strong positive correlations observed among intrinsic job satisfaction, extrinsic job satisfaction, overall job satisfaction, and work environment are also consistent with the interconnected nature of nurses’ workplace experiences. However, these correlations should be interpreted as associations rather than evidence that improvement in one construct causes improvement in another. The particularly strong correlations between overall job satisfaction and its intrinsic and extrinsic components are also expected because these dimensions contribute directly to the overall satisfaction construct. Therefore, the correlation findings provide supportive descriptive evidence of the relationships among the study variables but should not be interpreted as evidence of mediation or causal mechanisms.

Educational level was the only participant characteristic significantly associated with overall job satisfaction, whereas gender, age, and professional experience were not significantly associated with job satisfaction. Although this finding may indicate that educational background is relevant to nurses’ workplace experiences, it should be interpreted cautiously because the analysis was exploratory and the study was not specifically powered to examine demographic predictors of job satisfaction. The absence of significant associations for the other characteristics does not establish that these factors have no influence on job satisfaction. Rather, the findings suggest that the observed intervention effects were not readily explained by several commonly examined demographic characteristics.

### Strengths and limitations

This study has several strengths. First, the quasi-experimental pre-test and post-test control group design allowed changes over time to be examined in both intervention and control groups. Second, the study evaluated both job satisfaction and work environment, providing a broader assessment of nurses’ workplace outcomes than studies examining a single outcome. Third, baseline differences between groups were explicitly assessed and statistically addressed using ANCOVA. Fourth, the intervention was structured, delivered over a defined two-week period, and included multiple educational approaches rather than relying solely on didactic instruction. The subsequent observational monitoring of leadership behaviours over six months also provided an additional mechanism for assessing implementation of the program.

Several limitations should nevertheless be considered. First, the study was conducted in PHCs within a single geographic region of Saudi Arabia, which may limit the transferability of the findings to other regions, healthcare organizations, or countries. Second, the study used convenience non-probability sampling and the participating PHCs were not randomly allocated to intervention and control conditions. Consequently, selection bias and residual confounding cannot be excluded, particularly given the significant baseline differences observed across all four outcomes. Although ANCOVA was used to adjust for baseline scores, statistical adjustment cannot fully substitute for random allocation.

Third, the intervention was delivered to nurse managers at the PHC level, whereas the primary outcomes were measured among individual staff nurses. Thus, participants were situated within organizational and managerial clusters. The present analyses did not explicitly model clustering at the PHC or nurse-manager level, which may affect the precision of standard errors and statistical significance. Future studies should consider cluster-randomized designs or multilevel analytical approaches when the intervention is delivered at the managerial or facility level.

Fourth, the primary outcomes were based on self-reported questionnaires and may therefore have been influenced by response or social desirability bias. Blinding participants to intervention status was also not feasible given the nature of the leadership program. Finally, although observational follow-up of leadership behaviours continued for six months, the primary nurse outcomes were assessed immediately following the intervention. Therefore, the study cannot determine whether the observed improvements in job satisfaction and work environment were sustained over the longer term.

### Implications for practice

The findings have practical implications for nursing management and primary healthcare organizations. Structured transformational leadership development may be incorporated into professional development programs for nurse managers, particularly in settings undergoing organizational or health-system transformation. Rather than focusing exclusively on clinical competencies, leadership development can include communication, staff motivation, conflict management, ethical decision-making, teamwork, professional development, and individualized support for nurses.

For healthcare organizations in Saudi Arabia, such programs may complement broader workforce and quality-improvement initiatives under Saudi Vision 2030. However, leadership training should not be considered a substitute for addressing structural determinants of nurses’ work experiences, such as staffing, workload, resources, remuneration, and organizational policies. Leadership development is more likely to be effective when supported by an organizational environment that enables nurse managers to apply the behaviours acquired through training.

### Recommendations for further research

Future studies should use larger samples across multiple regions and healthcare organizations in Saudi Arabia to improve generalisability. Randomized or cluster-randomized designs would provide stronger evidence regarding the causal effects of transformational leadership programs. Because the intervention was implemented at the nurse-manager or PHC level, future studies should also use multilevel analytical approaches that account for clustering of nurses within managers and healthcare facilities.

Longer follow-up periods are needed to determine whether improvements in job satisfaction and work environment are sustained beyond the immediate post-intervention period. Future research could also examine potential mechanisms through which transformational leadership affects nurses’ outcomes, including psychological empowerment, work engagement, organizational commitment, perceived organizational support, and burnout. Qualitative research could further explore how nurses experience changes in managerial behaviours following leadership development programs and which components of the intervention are most influential.

## Conclusion

This study found that a structured transformational leadership program delivered to nurse managers was associated with significant improvements in staff nurses’ overall, intrinsic, and extrinsic job satisfaction and perceptions of the work environment in primary healthcare centers in Medina, Saudi Arabia. These differences remained significant after adjustment for baseline scores, supporting the potential effectiveness of transformational leadership development as an organizational strategy for improving nurses’ workplace experiences. However, the non-randomized design, convenience sampling, single-region setting, and reliance on self-reported outcomes warrant cautious interpretation of the findings. Integrating structured transformational leadership development into nurse manager training may provide a practical approach to strengthening supportive work environments and nurses’ workplace well-being in primary healthcare settings. Future multicenter studies using randomized or cluster-randomized designs and longer follow-up are needed to assess the effectiveness and sustainability of these effects.

## Ethical considerations

Ethical approval for this study was obtained from the Institutional Review Board (IRB), General Directorate of Health Affairs in Madinah, Ministry of Health, Saudi Arabia (National Registration Number with NCBE-KACST, KSA: H-03-M-84; IRB Log No. 25–082). The study received expedited ethical approval on 24 September 2025, prior to participant recruitment. The study procedures, including online data collection, informed consent process, data collection tool, and questionnaire, were conducted in accordance with the IRB-approved research protocol.

## Data Availability

The individual-level dataset underlying the results of this study is not publicly available because it contains demographic and occupational information collected from nurses participating in the study. Although direct personal identifiers have been removed, the combination of participant characteristics may present a risk of re-identification. The Institutional Review Board (IRB) approval explicitly requires that personally identifiable participant data must not be disclosed to any other party, that study data must be securely maintained for at least three years after completion of the study, and that the collected data should only be used for this research. Therefore, the participant-level dataset has not been deposited in a public repository. Requests for access to appropriately de-identified data may be submitted to the corresponding author at alrashidinasserayidhm@gmail.com. Requests should include the proposed purpose of data use and the requester’s institutional affiliation. Any request will be reviewed in accordance with the conditions of the ethical approval and applicable institutional requirements. Data will not be released where access would conflict with the ethical approval or participant confidentiality requirements. Figshare: Impact of a Transformational Leadership Programme for Nurse Managers on Nurses’ Job Satisfaction and Work Environment in Saudi Arabian Primary Healthcare Centres: A Quasi-Experimental Study: Questionnaire and Programme Training Schedule. https://doi.org/10.6084/m9.figshare.33109133.v1. ^14^ https://doi.org/10.6084/m9.figshare.33109133.v1 This project contains the following extended data:
• Questionnaire. Minnesota Satisfaction Questionnaire (MSQ) and Areas of Worklife Survey (AWS) used for data collection. • Program Training Schedule **.** Training schedule and educational materials for the transformational leadership programme. Data are available under the terms of the Creative Commons Attribution 4.0 International (CC BY 4.0) licence. Citation: Ayidh, M., & Alrashidi Nasser. (2026). Impact of a Transformational Leadership Programme for Nurse Managers on Nurses’ Job Satisfaction and Work Environment in Saudi Arabian Primary Healthcare Centres: A Quasi-Experimental Study: Questionnaire and Programme Training Schedule [Dataset]. Figshare. https://doi.org/10.6084/m9.figshare.33109133.v1 Figshare: TREND checklist for “Impact of a Transformational Leadership Program for Nurse Managers on Nurses’ Job Satisfaction and Work Environment in Saudi Arabian Primary Healthcare Centers: A Quasi-Experimental Study *.”* https://doi.org/10.6084/m9.figshare.33110084.
