# Supplementary material for: Impact of a Transformational Leadership Program for Nurse Managers on Nurses' Job Satisfaction and Work Environment in Saudi Arabian Primary Healthcare Centers: A Quasi-Experimental Study  
Source: F1000Res. 2026 Sep 10;15:1436. Originally published 2026 Aug 27. [Version 2] doi: 10.12688/f1000research.188950.2 (PMC13594605; doi:10.12688/f1000research.188950.2)
Supplement: Peer review history

# Reviewer report: Reviewer response for version 2

**DOI:** 10.5256/f1000research.209767.r520158

**Published:** 2026-09-21

**Authors:** Benny Hutahayan

**Affiliations:** 1 University of Brawijaya, Malang, East Java, Indonesia

I have reviewed the revised version of the manuscript and appreciate the authors’ careful attention to the comments and suggestions provided in the previous review. The authors have adequately addressed the points raised and have made appropriate revisions that improve the clarity, methodological presentation, and overall quality of the manuscript.

 The manuscript is now sufficiently clear and well presented, and I have no further major comments. Therefore, I am satisfied with the revisions and recommend the manuscript for approval in its current form.

Is the work clearly and accurately presented and does it cite the current literature?

Partly

If applicable, is the statistical analysis and its interpretation appropriate?

No

Are all the source data underlying the results available to ensure full reproducibility?

Partly

Is the study design appropriate and is the work technically sound?

No

Are the conclusions drawn adequately supported by the results?

Partly

Are sufficient details of methods and analysis provided to allow replication by others?

No

Reviewer Expertise:

Business Administration, Human Resources Management, Law, and Governance

I confirm that I have read this submission and believe that I have an appropriate level of expertise to confirm that it is of an acceptable scientific standard.

# Reviewer report: Reviewer response for version 1

**DOI:** 10.5256/f1000research.208540.r516076

**Published:** 2026-09-01

**Authors:** Suryanti Suryanti

**Affiliations:** 1 University of Batam, Batam, Indonesia

This manuscript reports a quasi-experimental pre-test/post-test study with a control group evaluating the impact of a structured transformational leadership program delivered to nurse managers on nurses’ job satisfaction and perceptions of the work environment in primary healthcare centers in Medina, Saudi Arabia.

 The study included 104 nurses from seven Ministry of Health primary healthcare centers, with 52 nurses assigned to the intervention group and 52 to the control group. The intervention consisted of a structured transformational leadership program delivered to nurse managers over two weeks and included interactive lectures, group discussions, case-based learning, role-playing, leadership simulations, and practical problem-solving activities.

 Nurses’ job satisfaction was assessed using the Minnesota Satisfaction Questionnaire (MSQ), while perceptions of the work environment were assessed using the Areas of Worklife Survey (AWS). The study used pre- and post-intervention assessments, with paired-samples t-tests used to examine within-group changes and ANCOVA used to compare post-intervention outcomes between groups while adjusting for baseline scores.

 The study reports significant improvements in overall, intrinsic, and extrinsic job satisfaction and work environment scores in the intervention group. The adjusted ANCOVA analyses also demonstrated statistically significant differences between the intervention and control groups for all four outcomes, with large reported effect sizes.

 Overall, the manuscript addresses a relevant topic in nursing management and primary healthcare. The study is clearly presented, the research question is appropriate, the methodology is sufficiently described, and the conclusions are generally consistent with the reported findings. The authors also acknowledge the principal methodological limitations of the study, including the non-randomized design, convenience sampling, single-region setting, self-reported outcomes, and the absence of clustering in the statistical analysis.

 Overall assessment

 I found the manuscript to be clearly written and logically structured. The introduction provides an appropriate rationale for examining transformational leadership as an organizational intervention, and the literature cited provides relevant background on transformational leadership, nurses’ job satisfaction, and the nursing work environment. The manuscript also includes recent literature, including systematic and integrative reviews and recent intervention research.

 The study design is appropriate for the research question. Although the study is not randomized, the inclusion of a control group and pre-intervention measurements provides a useful basis for assessing changes associated with the intervention. The authors appropriately identified baseline differences between the intervention and control groups and incorporated baseline scores into the subsequent ANCOVA analyses.

 The description of the intervention is also a strength. The manuscript specifies the duration, training structure, content, and educational approaches used in the transformational leadership program. The availability of the questionnaire and programme training schedule as extended data further supports transparency and reproducibility.

 The results are presented clearly in Tables 1–6, and the discussion appropriately relates the findings to previous literature. Importantly, the authors do not appear to overstate the findings in the discussion. They explicitly recognize that the study design does not allow specific causal pathways to be established and appropriately interpret the correlation analyses as associations rather than causal relationships.

 The limitations are also appropriately acknowledged. In particular, the authors recognize that the participating PHCs were not randomly allocated, convenience sampling was used, and the study was conducted in a single geographic region. They also explicitly acknowledge that nurses were clustered within PHCs/nurse managers and that clustering was not incorporated into the present statistical analyses.

 Statistical analysis

 I am not qualified to provide a definitive assessment of the statistical analysis and therefore recommend that the statistical component of the manuscript be reviewed by a qualified statistician.

 In particular, statistical review would be useful because the intervention was delivered at the nurse-manager/primary healthcare center level, whereas the outcomes were measured at the individual nurse level. Consequently, observations may be clustered within healthcare centers and nurse managers. The authors have appropriately recognized this issue as a limitation and recommend multilevel analytical approaches or cluster-randomized designs in future research.

 A qualified statistician may therefore wish to verify the appropriateness of the analytical approach, including the ANCOVA model, assumptions underlying the analyses, and the reported effect sizes and significance levels in view of the hierarchical structure of the data.

 This point is raised as a recommendation for statistical verification rather than as a conclusion that the reported findings are invalid. The remaining methodological and interpretative aspects of the manuscript appear reasonable based on the information provided.

 Data availability and reproducibility

 The manuscript provides an appropriate explanation for why individual-level data are not publicly deposited, citing participant confidentiality and the requirements of the relevant ethical approval. The authors nevertheless provide a mechanism for requesting appropriately de-identified data, subject to ethical and institutional requirements.

 The extended data also include the questionnaires and the programme training schedule, which are useful for understanding and reproducing the intervention and outcome assessment procedures.

 Conclusions

 The conclusions are adequately supported by the results presented. The intervention group showed improvements in all four assessed outcomes, and the differences remained statistically significant after adjustment for baseline scores.

 The conclusion is appropriately cautious in acknowledging the non-randomized design, convenience sampling, single-region setting, self-reported outcomes, and need for longer-term follow-up.

Is the work clearly and accurately presented and does it cite the current literature?

Yes

If applicable, is the statistical analysis and its interpretation appropriate?

I cannot comment. A qualified statistician is required.

Are all the source data underlying the results available to ensure full reproducibility?

Yes

Is the study design appropriate and is the work technically sound?

Yes

Are the conclusions drawn adequately supported by the results?

Yes

Are sufficient details of methods and analysis provided to allow replication by others?

Yes

Reviewer Expertise:

healthcare qualitu assurance, patient safety, medical education, public health

I confirm that I have read this submission and believe that I have an appropriate level of expertise to confirm that it is of an acceptable scientific standard.

## References

[1] Des Jarlais DC, Lyles C, Crepaz N; TREND Group. Improving the reporting quality of nonrandomized evaluations of behavioral and public health interventions: the TREND statement. Am J Public Health. 2004;94(3):361-366.10.2105/ajph.94.3.361PMC144825614998794

# Reviewer report: Reviewer response for version 1

**DOI:** 10.5256/f1000research.208540.r516077

**Published:** 2026-09-01

**Authors:** Benny Hutahayan

**Affiliations:** 1 University of Brawijaya, Malang, East Java, Indonesia

The Introduction states that most previous studies were observational or correlational in nature and that evidence from intervention-based studies in Saudi Arabian primary health care (PHC) remains limited. However, this gap is still described in general terms. The authors need to explain more explicitly what remains unknown from previous research and how this study specifically fills that gap.

 There is no chapter on literature review and hypothesis development. The authors need to add one.

 The manuscript explains that extrinsic satisfaction consists of 6 items on a 1–5 scale, so theoretically the maximum score is 30. However, the study results report a post-intervention average extrinsic satisfaction score of 38.06. The authors need to explain which version of the MSQ was used, the item breakdown, the score range, and the calculation method.

 The number of nurse managers who participated in the program, their characteristics, the number of nurses under each manager, and the distribution of participants across each PHC should be reported.

 The manuscript should list all items used, the response scale, the scoring method, and the score range.

 The statement that the content validity index meets an acceptable threshold is still too general. The authors should include the results of their validity tests in the manuscript.

 The manuscript states that the homogeneity of regression slopes was examined, but the results of this examination are not presented. The authors should report the results of their assumption tests.

 The manuscript places heavy emphasis on p-values and effect sizes. Confidence intervals would help readers assess the precision of the reported intervention effects.

 The discussion states that recognition, encouragement, professional development, and participation can explain the increase in satisfaction. However, these mediating variables were not measured. The author should be more careful when discussing the research findings. Please double-check the entire discussion chapter.

Is the work clearly and accurately presented and does it cite the current literature?

Partly

If applicable, is the statistical analysis and its interpretation appropriate?

No

Are all the source data underlying the results available to ensure full reproducibility?

Partly

Is the study design appropriate and is the work technically sound?

No

Are the conclusions drawn adequately supported by the results?

Partly

Are sufficient details of methods and analysis provided to allow replication by others?

No

Reviewer Expertise:

Business Administration, Human Resources Management, Law, and Governance

I confirm that I have read this submission and believe that I have an appropriate level of expertise to confirm that it is of an acceptable scientific standard, however I have significant reservations, as outlined above.

# Response

**Published:** 2026-09-01

**Authors:** Alrashidi Nasser Ayidh M

**Affiliations:** Lincoln University College, Petaling Jaya, Selangor, Malaysia

**Reviewer: **The Introduction states that most previous studies were observational or correlational in nature and that evidence from intervention-based studies in Saudi Arabian primary health care (PHC) remains limited. However, this gap is still described in general terms. The authors need to explain more explicitly what remains unknown from previous research and how this study specifically fills that gap.

**Response: **Thank you for this important comment. We have revised the Introduction to clarify the specific evidence gap. The revised manuscript now distinguishes between evidence describing associations between transformational leadership and nurses’ outcomes and the limited evidence from structured leadership development interventions. We have explicitly stated that it remains unclear whether a structured transformational leadership programme delivered to nurse managers can improve staff nurses’ job satisfaction and perceptions of the work environment in primary healthcare settings in Saudi Arabia. We have also clarified how the present quasi-experimental study addresses this gap by evaluating these outcomes following a structured intervention in primary healthcare centres in Medina, Saudi Arabia.

**Reviewer: **There is no chapter on literature review and hypothesis development. The authors need to add one.

**Response: **Thank you for this suggestion. We have added a new section entitled “Literature Review and Hypothesis Development” to provide a more explicit synthesis of the relevant literature and the theoretical rationale for the study. The new section discusses the relationship between transformational leadership, nurses’ job satisfaction, and the work environment and provides the basis for the study hypotheses.

**Reviewer: **The manuscript explains that extrinsic satisfaction consists of 6 items on a 1–5 scale, so theoretically the maximum score is 30. However, the study results report a post-intervention average extrinsic satisfaction score of 38.06. The authors need to explain which version of the MSQ was used, the item breakdown, the score range, and the calculation method.

**Response: **Thank you for identifying this important issue. We have clarified the MSQ description in the Methods section. The revised manuscript specifies that the study used a 20-item questionnaire based on the short-form Minnesota Satisfaction Questionnaire (MSQ), comprising 12 items for intrinsic job satisfaction and 8 items for extrinsic job satisfaction. Each item was scored on a five-point scale from 1 to 5. Intrinsic satisfaction therefore has a possible score range of 12–60, extrinsic satisfaction 8–40, and overall job satisfaction 20–100. Scores were calculated by summing the corresponding item scores. This clarification explains the reported post-intervention extrinsic satisfaction score of 38.06.

**Reviewer: **The number of nurse managers who participated in the program, their characteristics, the number of nurses under each manager, and the distribution of participants across each PHC should be reported.

**Response: **Thank you for this comment. We have expanded the Participants and Sample Size section to report that the transformational leadership programme was delivered to 52 nurse managers in the intervention PHCs. We have also reported the distribution of the 104 participating nurses across the seven PHCs: Al-Hijrah (n = 16), Wairah (n = 11), Al-Safiyyah (n = 13), Health Surveillance Center (n = 12), Al-Daithah (n = 13), Bab Jibreel (n = 14), and Al-Salsalah (n = 15).

 The available study dataset did not include individual demographic and professional characteristics of the nurse managers, and individual nurse–manager linkages were not recorded. We have therefore stated transparently in the revised manuscript that manager-level characteristics and the number of nurses supervised by each individual manager could not be determined retrospectively.

**Reviewer: **The manuscript should list all items used, the response scale, the scoring method, and the score range.

**Response: **Thank you. We have expanded the description of both outcome instruments in the Methods section. The revised manuscript now specifies the number of items, domains, response scales, scoring procedures, and possible score ranges for both job satisfaction and work environment measures. The full set of questionnaire items used in the study is also provided as Extended Data.

**Reviewer: **The statement that the content validity index meets an acceptable threshold is still too general. The authors should include the results of their validity tests in the manuscript.

**Response: **Thank you for this valuable comment. We have revised the instrument validation section to report the numerical results of the content validity assessment. Specifically, the item-level content validity index (I-CVI) ranged from 0.83 to 0.94 across the questionnaire items, indicating acceptable content validity. This information has now been explicitly reported in the Methods section.

**Reviewer: **The manuscript states that the homogeneity of regression slopes was examined, but the results of this examination are not presented. The authors should report the results of their assumption tests.

**Response: **Thank you for pointing this out. We have clarified in the Statistical Analysis section that the assumption of homogeneity of regression slopes was assessed by testing the interaction between study group and the corresponding baseline outcome score before conducting ANCOVA.

 The Results section now reports the assumption-test results. The group-by-baseline interaction was not statistically significant for overall job satisfaction (F = 0.948, p = 0.333), intrinsic job satisfaction (F = 2.931, p = 0.090), extrinsic job satisfaction (F = 0.200, p = 0.656), or work environment (F = 0.569, p = 0.452), supporting the assumption for all four outcomes.

**Reviewer: **The manuscript places heavy emphasis on p-values and effect sizes. Confidence intervals would help readers assess the precision of the reported intervention effects.

**Response: **We agree with the reviewer. To improve the reporting of the intervention effects, we have added 95% confidence intervals for the adjusted mean differences to the ANCOVA results table. The Results section has also been revised to report the adjusted mean differences together with their 95% confidence intervals, *p*-values, and partial η² values.

**Reviewer: **The discussion states that recognition, encouragement, professional development, and participation can explain the increase in satisfaction. However, these mediating variables were not measured. The author should be more careful when discussing the research findings. Please double-check the entire discussion chapter.

**Response: **Thank you for this important observation. We have carefully revised the Discussion to ensure that mechanisms that were not directly measured are not presented as demonstrated mediators or causal pathways. The revised Discussion now distinguishes between the theoretical mechanisms described in the transformational leadership literature and the outcomes actually measured in this study. We have explicitly acknowledged that specific processes such as communication, recognition, participation, professional development, and other potential mechanisms were not directly assessed.

 We have also clarified that the observed intervention effects represent adjusted differences between groups and do not establish specific causal pathways. In addition, a limitation has been added to acknowledge that potential mechanisms underlying the observed effects were not measured. The correlation findings are likewise explicitly described as associations rather than evidence of mediation or causality.

 We sincerely thank the reviewer for the constructive and detailed comments. We believe that these revisions have substantially improved the methodological transparency, statistical reporting, interpretation, and overall clarity of the manuscript. All changes have been made in the revised manuscript with Track Changes enabled for ease of review.
